# Beyond the tail: the consequence of context in histone post-translational modification and chromatin research

**DOI:** 10.1042/BCJ20230342

**Published:** 2024-02-14

**Authors:** Ellen N. Weinzapfel, Karlie N. Fedder-Semmes, Zu-Wen Sun, Michael-Christopher Keogh

**Affiliations:** EpiCypher Inc., Durham, NC 27709, U.S.A.

**Keywords:** chromatin, histone code, histone peptides, histone post-translational modifications, histones, nucleosome

## Abstract

The role of histone post-translational modifications (PTMs) in chromatin structure and genome function has been the subject of intense debate for more than 60 years. Though complex, the discourse can be summarized in two distinct — and deceptively simple — questions: What is the function of histone PTMs? And how should they be studied? Decades of research show these queries are intricately linked and far from straightforward. Here we provide a historical perspective, highlighting how the arrival of new technologies shaped discovery and insight. Despite their limitations, the tools available at each period had a profound impact on chromatin research, and provided essential clues that advanced our understanding of histone PTM function. Finally, we discuss recent advances in the application of defined nucleosome substrates, the study of multivalent chromatin interactions, and new technologies driving the next era of histone PTM research.

## Introduction

Chromatin refers to the molecular packaging of DNA inside eukaryotic cells. The basic repeating subunit is the nucleosome: a histone octamer (two H2A/H2B dimers and one H3/H4 tetramer) wrapped with ∼147 bp DNA. Though originally considered a simple packaging system for DNA, further investigation revealed nucleosomes to be a highly dynamic structure central to genome regulation [1]. As a result, chromatin impacts most biological processes, including cell division, apoptosis, differentiation, and disease development [2–6].

Bulk chromatin is distinguished by various modifications and protein-protein interactions that alter accessibility to DNA, create permissive/repressive domains, and denote specific genomic features (e.g. promoters). *DNA methylation* is catalyzed by DNA methyltransferases and is generally associated with repressive chromatin (not discussed here; for review, see [7–10]). *Histone post-translational modifications (PTMs)* are chemically diverse, covalent protein modifications, including methylation, acetylation, phosphorylation, ubiquitination, ADP ribosylation. These are primarily catalyzed on the extended histone ‘tails,’ although residues within the histone globular core can be targeted. Chromatin also interacts with a broad array of *chromatin-associated proteins*: transcription factors, chromatin-modifying enzymes, nucleosome remodelers, and various adapters, cofactors, or other binding proteins. The enzymes that add PTMs are termed *writers*, while those that remove are *erasers*. Histone PTMs can alter nucleosome electrostatic charge, changing nucleosome conformation and DNA accessibility, and/or be ‘read’ by specific chromatin-associated proteins, instructing downstream events. These *reader proteins* detect specific histone PTMs and often act as scaffolding to recruit additional chromatin regulators.

How do these various structural elements orchestrate chromatin function? Early studies by C. David Allis and others linked histone PTMs to gene expression, leading to an explosion of work on writers, readers, and erasers. Additional research revealed direct roles for histone PTMs in DNA double-strand break responses [11], meiotic recombination [12], and autoimmune disease [13]. Yet, *how* we study the modifications has become of equal importance, and a topic of continued debate. For robust activity, chromatin regulators often require multiple binding events, involving histone PTMs, DNA, the nucleosome core, and other protein subunits: a concept known as *multivalency*. Dissecting this complexity demands highly defined, physiologically relevant experimental strategies.

*Progress in science depends on new techniques, new discoveries and new ideas, probably in that order*. Sydney Brenner (1927–2019; 2002 Nobel Prize in Physiology or Medicine)

**Figure 1.**
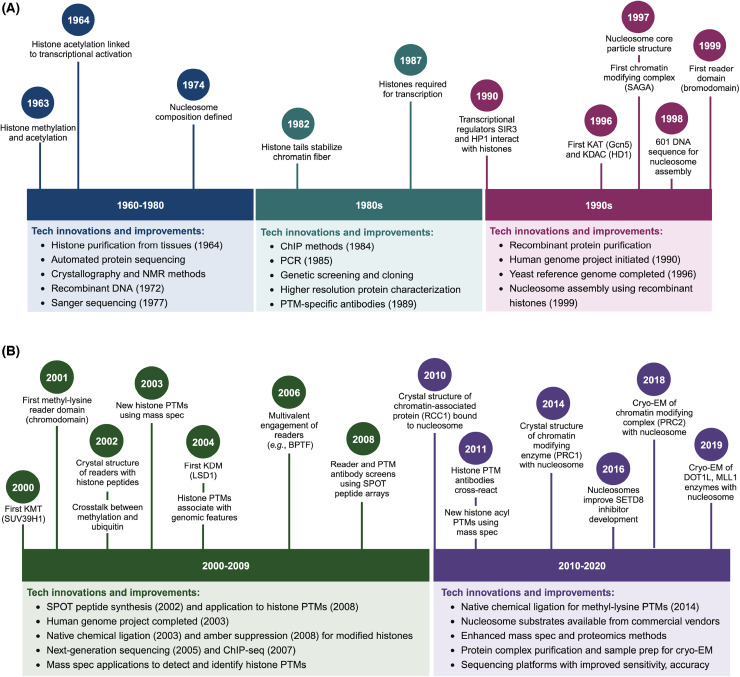
Our increasing understanding of chromatin biology, nucleosome structure, and histone PTM function was driven by continued technological innovation and improvement. (**A**) Pre-2000. (**B**) 2000–2020. Key discoveries are denoted by year and specific innovations within each time period noted. Key: ChIP, chromatin immunoprecipitation; KAT, lysine acetyltransferase; KDAC, lysine deacetylase; NMR, nuclear magnetic resonance, cryo-EM, cryogenic electron microscopy; KDM, lysine demethylase; KMT, lysine methyltransferase. Figure created with BioRender.

Sydney Brenner, a dominant figure in the history of molecular and developmental biology, frequently discussed how invention — new methods, equipment, reagents, etc. — is critical to scientific discovery [14]. As such, our growing understanding of chromatin function parallels innovations in biomedical research technologies (Figure 1). In this review, we provide a perspective on how chromatin-based reagents and methodologies have shaped the field of epigenetics research. We describe the challenges of each era, how technological innovations enabled continuous gains in insight, and discuss recent studies that illustrate the necessity of nucleosome context for chromatin research. We end on some questions that remain, whether we can address them with our current capabilities, and future areas of scientific growth.

## Linking histone PTMs to transcription

Histone proteins were discovered by Albrecht Kossel in 1884, as part of his Nobel prize winning work on the chemical composition of nucleic acids [15]. Early characterization suggested species- and cell-type specific histones, driving hypotheses about their involvement in gene expression [16,17]. However, these ideas gained little traction until validation of the chromosome theory of inheritance and publication of the double helical structure of DNA. Histones were known to bind DNA and repress *in vitro* transcription by the early 1960s [18], but the discovery that they can be methylated [19] and acetylated [20] prompted additional questions about function.

### Early characterization of nucleosomes and histone PTMs

Vincent Allfrey's discovery that histone acetylation supports gene expression, without evicting histones from DNA, was an important step in our understanding of chromatin biology [21]. His later work would show that acetylation is enriched on the N-terminal tails of histone H3 and H4 [22,23]. The acetylation sites are highly conserved from plants to mammals [24,25], suggesting critical roles across evolution. Allfrey theorized that lysine acetylation on the histone tails neutralizes their positive charge, reducing associations with negatively charged DNA, thus supporting transcriptional activation. However, this concept was widely contested due to the varying effects of histone acetylation on [histone : DNA] affinity *in vitro* [26–30]. Furthermore, such a model did not adequately explain histone methylation, as this PTM class does not alter charge.

Unfortunately, Allfrey's theory of histone PTM function was difficult to prove using contemporary techniques. Indeed, up through the 1990s researchers used histones extracted from animal tissues, such as calf thymus, or eukaryotic cell lines, most notably HeLa. Histone purification from these sources required acid or ethanol extraction protocols, which were notoriously difficult, imprecise, and time-consuming [31–33]. Individual histones were isolated by protein fractionation and chromatography [34], but yields and quality varied widely due to denaturing purification conditions and/or contaminating proteases. The resulting histones were also highly heterogeneous, with PTM profiles that could differ widely across experiments [31–33]. These issues made it difficult to study [histone : DNA] interactions, and next to impossible to study the function of individual PTMs.

Instead, research in the 1960s and 1970s focused on the role of histones in chromatin architecture, folding, and stability. Why were there so many different histone proteins, and how did they function together on DNA? Roger Kornberg utilized enhanced X-ray diffraction to define nucleosome composition in 1974 [35,36], and in the same year, Olins and Olins [37] defined the classic ‘beads-on-a-string’ chromatin model. The apparent uniformity of nucleosomes [35–37], combined with their broad genomic distribution, led many scientists to conclude that chromatin is a simple packaging structure for DNA, with nonspecific roles in gene expression [38,39]. Due to this consensus, and a lack of suitable tools, histone PTMs were generally ignored during this period.

### Advances in chromatin substrates and structural techniques reveal ‘fuzzy’ histone tails

The advent of recombinant DNA and molecular cloning, combined with improved protein purification and X-ray crystallography methods, allowed huge advances in the study of nucleosome structure (Figure 1A). Researchers could now generate pure, unmodified histones, without the need for complex acid extractions. In 1997, Karolin Luger with Tim Richmond and colleagues unified these capabilities to create a high-resolution crystal structure of the nucleosome [40–42]. Preferred DNA wrapping sequences were defined by the Widom group in 1998 [43], supporting more efficient nucleosome assembly.

The 1997 crystal structure uncovered a central regulatory feature of the nucleosome: the *acidic patch*. This negatively charged region on the surface of nucleosome globular core, at the interface of H2A and H2B [40], established strong contacts with the highly basic H4 tails. In the structure, H4 tail acetylation was predicted to disrupt its binding to the nucleosome core, supporting previous *in vitro* experiments [44]. Later studies would show that [H4 : acidic patch] interactions are critical for chromatin compaction and stability [45–48], hinting at the importance of nucleosome context in chromatin research.

Other than these H4 interactions, the highly dynamic N-terminal histone tails were difficult to capture by X-ray diffraction, and the crystal structure could not provide definitive insight [40]. Without knowing their approximate position relative to the octamer core and nucleosomal DNA, scientists relied on protease digestion [49] and histone peptide based methods to determine potential functions. The results were confusing, and often contradictory. For instance, although histone tails bind nucleosomal DNA in solution [50–52] and can directly impact transcription factor binding [44], they are not necessary for nucleosome assembly, nor do they impact nucleosome stability [53]. Yet, histone tails are required for the *in vitro* assembly of chromatin fibers [54], suggesting potential inter-nucleosomal interactions.

**Figure 2.**
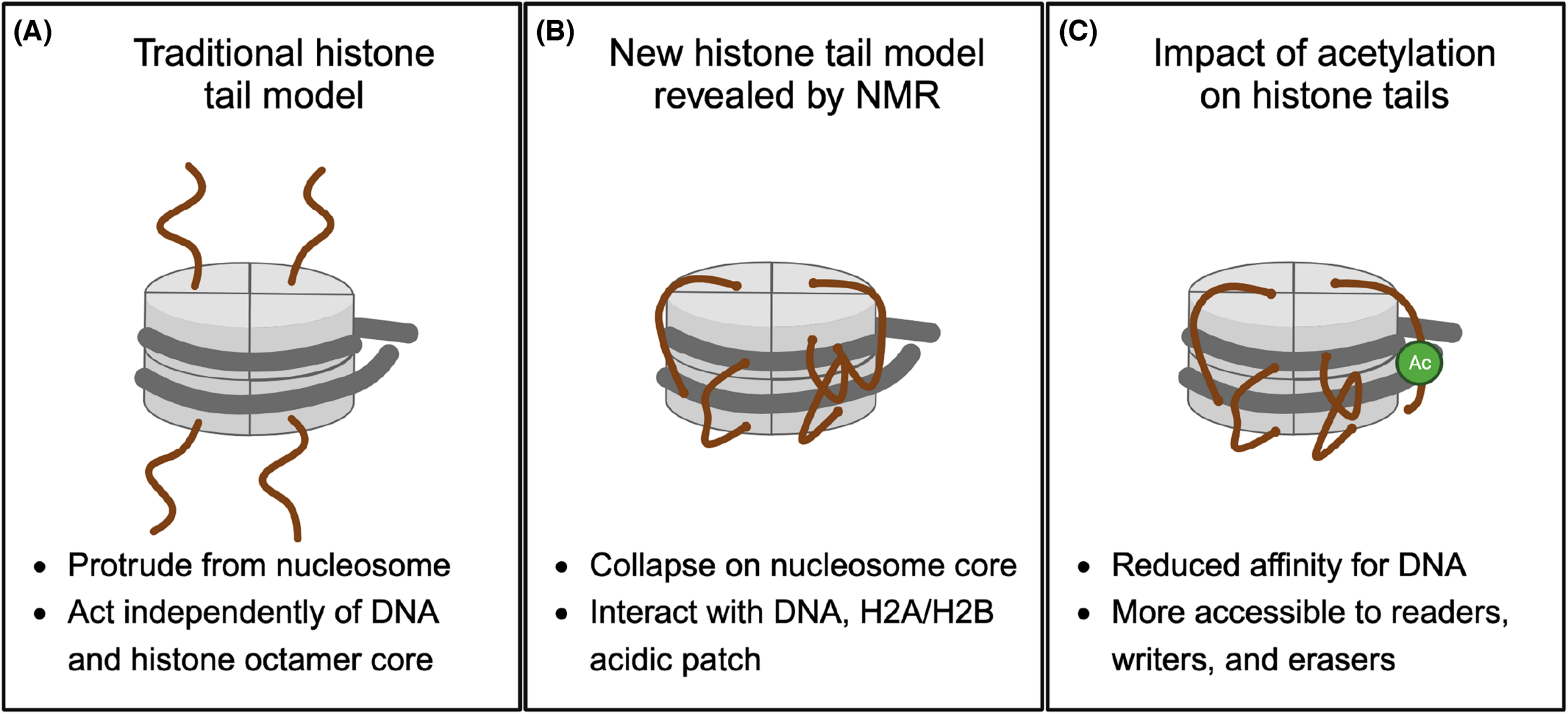
The histone tails make extensive (and regulatable) contacts with the nucleosome core particle. (**A**) Original crystal structures of the nucleosome [40] could not visualize N-terminal tails, leading many to assume they protrude from the core into solution [55,56]. (**B**) However, NMR shows that unmodified, positively-charged histone tails collapse onto the nucleosome surface, and make extensive contacts with negatively charged DNA and the H2A/H2B acidic patch [57,58]. (**C**) Acetylation neutralizes tail positive charge and disrupts these interactions, highlighting the role of nucleosome electrostatics in chromatin interactions [57,59]. Other PTMs that modulate charge include the larger family of lysine acylations [60], serine and threonine phosphorylation [61], or arginine deimination/citrullination [62]. Figure created with BioRender.

Despite much evidence to the contrary, the nucleosome model that emerged from this time showed histone tails extended outward from the nucleosome core (Figure 2A). Nucleosomes were considered essential for packaging DNA and represented roadblocks to transcription factor binding, while histone acetylation supported DNA accessibility. As mentioned above, the ostensible uniformity of nucleosome structure and the lack of site-specific histone modifying enzymes led many to assume minor roles for histones and histone PTMs in gene regulation [63].

### Model organisms link transcriptional activation to histone modifying enzymes

Emerging molecular biology techniques, such as PCR and Sanger sequencing, helped advance the study of transcriptional regulation (Figure 1A). The budding yeast *Saccharomyces cerevisiae* proved an essential tool for the field, owing to its haploid genome, defined cell/reproductive cycles, ease of culturing, and flexibility for genetic manipulation/phenotypic selection. Mutant screens for loss or gain of function in novel transcriptional regulators identified histones [64,65], as well as proteins eventually linked to histone PTMs and chromatin structure: SWI/SNF chromatin remodelers [65–67], heterochromatin-related SIR proteins [68–70], components of the SAGA transcriptional co-activator complex [71–75], and many more [76]. Of particular note, Fred Winston and Michael Grunstein developed genetic tools to alter yeast histone protein levels [77–80], establishing the importance of nucleosomes for transcription *in vivo* and revealing novel interactions between histones and transcriptional regulators [80–83]. The mutation of histone residues to prevent or mimic their modification (e.g. K to R creates an unmodifiable charge while K to Q mimics charge neutralization) also suggested roles for lysine acetylation in chromatin interactions [81–83].

To gain further insight, Allis had developed a new model organism: the protozoan ciliate *Tetrahymena thermophilia*. In each cell, *Tetrahymena* maintains a transcriptionally active somatic genome (macronucleus) and a repressed germline genome (micronucleus) [84], and thus proved invaluable for studying the relationship between histone PTM state and gene expression. Using this model system, the Allis group worked for nearly a decade characterizing histone acetyltransferase or HAT (now more accurately termed lysine acetyltransferase, *KAT* [85]) activity, leading to purification of the first active enzyme in 1995: a biochemical *tour de force* [86]. In 1996, they successfully isolated the encoding *Tetrahymena* gene [87] and showed it targeted specific H3 and H4 residues [88]. This protozoan KAT also had a yeast homolog: the extensively studied transcriptional activator Gcn5. Notably, Gcn5 interacted with other transcriptional activators [89,90] and was highly conserved from yeast to human [73], but its precise function had been unclear. Thus, Allis and colleagues had simultaneously identified the first KAT, showed that it modifies distinct histone sites, and established a clear mechanistic link between histone acetylation and gene activation: a field-exploding breakthrough.

A mere 2 months after the publication of the first KAT, the Schreiber lab reported the first histone deacetylating enzyme (mammalian HD1), that was related to a yeast transcriptional corepressor (Rpd3) [91]. Combined, these studies confirmed the importance of histones and their modifying enzymes in gene expression. As per Allis ‘It couldn't have been a more wonderful one-two punch. I am not sure that the chromatin field has been the same since’ [92].

## Establishing the ‘histone code’ hypothesis: a PTM-based chromatin language

Site-specific histone modifying enzymes had been discovered, but the underlying cause and consequence of their specificity was still poorly understood. A major discovery came in 1999, when it was determined that the P/CAF bromodomain — a highly conserved module and standard feature of KAT transcriptional co-activator complexes — specifically binds acetylated histones (Figure 1A) [93]. This *reader* inspired new questions about histone PTMs and their potential to recruit chromatin-modifying enzymes and remodeling complexes.

**Figure 3.**
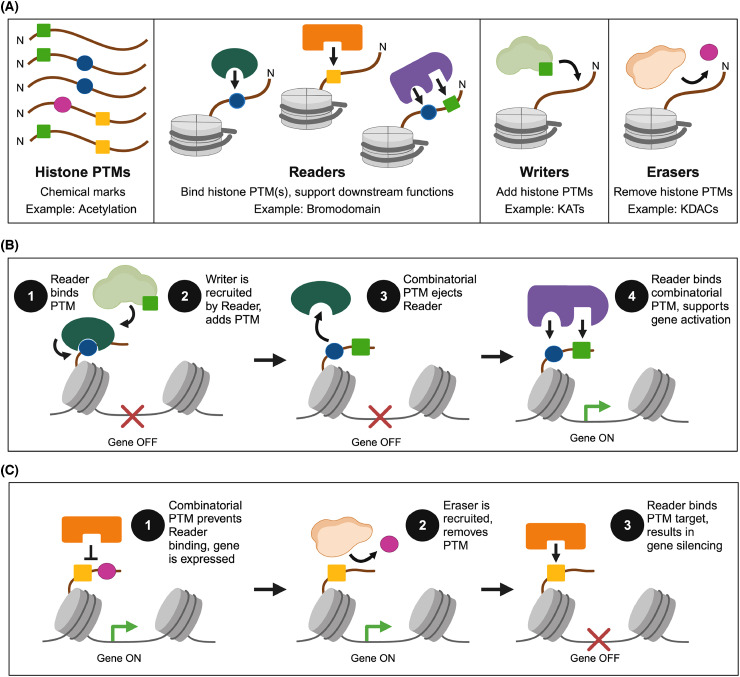
Potential mechanisms and functions of combinatorial histone PTMs as outlined in the original ‘histone code’ hypothesis [61]. (**A**) Key elements of the language. (**B,C**) Examples of readout include where sequential histone PTMs facilitate gene activation (**B**), or combinatorial histone PTMs regulate gene activation (**C**). Figure created with BioRender.

In 2000, Strahl and Allis hypothesized the *histone code*, where histone PTMs act as a language read by proteins to regulate chromatin structure, transcription, and other molecular processes [61]. The code was defined by combinatorial histone PTMs, or unique combinations of PTMs, on the same or different histone tails, to modulate chromatin interactions. Strahl and Allis discussed how the code may enable site specificity for chromatin-modifying enzymes, similar to the structural recognition motifs of kinases. Potential combinatorial mechanisms (Figure 3) included sequential histone modification pathways, in which one PTM recruits or blocks additional modifying enzymes; cooperative engagement of proteins/enzymes, to help increase enzyme efficiency; and situations where PTMs act in *trans* (i.e. on separate histone tails) to form unique binding sites.

Importantly, the histone code was more than a binary on-off system, in which PTMs are the only factors regulating transcription. Strahl and Allis proposed that PTMs are deposited in a site-specific manner to help recruit or repress the binding of other chromatin interactors, such as transcription factors or chromatin-modifying enzymes, thereby contributing to transcriptional regulation [61,94]. It did not intend to place histone modifications as the master regulator of all gene regulatory pathways, but rather aimed to show how histone PTMs could facilitate specific gene expression programs. This was a striking departure from the prevailing opinion that PTMs impacted transcription only indirectly, by altering nucleosomal charge and disrupting DNA-histone or histone-histone interactions. Yet (and as per Strahl and Allis), if this were the case, one would expect a few PTMs with easily predictable impacts on chromatin structure. Instead, the number and types of chromatin modifications were rapidly expanding, and already encompassed methylation, acetylation, phosphorylation, and ubiquitination, indicating a high level of complexity. Below we highlight discoveries from 2000 to 2010 that provided support for the histone code hypothesis as it drove a PTM-focused era of chromatin research (Figure 1B).

### Advances in histone lysine methylation support the histone code

The Strahl and Allis article on a PTM code came at the perfect time, preceding major studies on histone methylation that would support its existence (Figure 3). In 2000 the Jenuwein and Allis groups defined the first histone lysine methyltransferase (*KMT*): SUV39H1, which specifically methylates H3K9 to establish silenced heterochromatin domains [95]. Of perhaps greatest impact, this work mapped SUV39H1 enzyme function to its SET domain, which has homologs in multiple proteins and species. Uncovering the representative function of a highly conserved domain opened the door to a remarkable era of chromatin discovery.

Were histone lysine methylation PTMs also read, just as bromodomains read acetylation marks? The exploration of heterochromatin proteins soon provided an answer. HP1 was first described in *Drosophila melanogaster* [96], where it plays key roles in heterochromatin stability and gene silencing [97,98]. Analysis of its protein sequence led to identification of the chromodomain (CD), a highly conserved 40–50 amino acid region shared by many chromatin-associated proteins. HP1 localization and gene silencing activity required an intact CD and SUV39H1 expression. However, the HP1 CD was not known to interact with SUV39H1, nor did it bind a specific DNA motif, challenging mechanistic analysis. The discovery of SUV39H1 KMT activity provided essential context, and in 2001 three groups simultaneously showed that the HP1 CD binds H3K9me2/me3: the first methyl-lysine reader [99–101]. The resulting insight to heterochromatin function involved KMTs, histone PTMs, and readers, demonstrating the inherent connections between different chromatin regulatory proteins [94].

The study of KMTs also led to the investigation of histone monoubiquitination (Kub1), which illustrated how proteins leverage multiple enzymatic and/or binding domains to engage in epigenetic cross-talk. Ubiquitination is catalyzed by E3 ligases, and is most widely known for the role of polyubiquitination in protein degradation. However, the discovery of unique receptors that bind Kub1 to modulate signaling pathways, gene expression, and the DNA damage response, led scientists to reconsider the potential function of this PTM on chromatin [102]. In 2002, several groups showed that H2BK120ub1 was required for methylation of H3K4 [103,104] and H3K79 [105,106]. Subsequent studies identified H2BK120ub1 and H2AK119ub1 as central components of multiple KMT pathways, respectively regulating euchromatin and heterochromatin function [107].

With every passing year, research on the repressive Polycomb group (PcG) proteins uncovered increasingly intricate modalities of gene regulation. PcG proteins, including EZH2, were first identified in *Drosophila* as antagonistic regulators of *HOX* genes during embryonic development, and linked to heterochromatin via studies of position-effect variegation [108]. EZH2 was among the original SET domain proteins, alongside SUV39H1, but curiously did not exhibit methylation activity on histone peptides [95]. Did EZH2 possess KMT activity, and could this contribute to gene repression? In 2002, multiple groups converged on the discovery of polycomb repressive complex 2 (PRC2), showing that EZH2 requires association with subunit EED for catalytic activity [109–111]. EZH2 catalyzed the H3K27 mono-, di- and tri- methyl states, with a strong preference for nucleosome substrates [109–111]; illustrating the importance of context for chromatin regulators.

Amidst this tumult was years of debate on the reversibility of histone lysine methylation. The field remained uncertain until the discovery of the first lysine demethylase (*KDM*), LSD1, by Yang Shi in 2004 [112]. Similar to other chromatin-modifying enzymes, LSD1 showed high evolutionary conservation (e.g. *S. pombe*, *Drosophila*, mouse) and displayed site-specific enzymatic activity. However, *Schizosaccharomyces cerevisiae* did not contain any immediate homologs, despite lysine mono-, di-, and tri-methylation at H3K4, H3K36 and H3K79. Furthermore, LSD1 enzymology could only accommodate H3K4me1 and H3K4me2; how was the tri-methyl being removed? At around the same time, Yi Zhang and colleagues had developed an unbiased screening approach to identify new KDMs in HeLa cell extracts. Their seminal work uncovered the first Jumonji C (JmjC) histone lysine demethylase: JHDM1/KDM2 [113]. JmjC domains use an oxidative demethylation mechanism, which can target mono-, di-, and tri-methyl states: so now all methylation states were potentially reversible. This domain was highly conserved across eukaryotes, including *S. cerevisiae*, and has revealed many chromatin-modifying enzymes, disease-relevant pathways, and cancer drug targets [114].

### An explosion of histone PTMs and regulators reveal connections to disease

Post-2000, the number of histone PTMs, modifying enzymes, and reader proteins expanded at a breathtaking rate (Table 1). Newly defined chromatin-modifying complexes (e.g. NURF, PRC2) often contained multiple reader domains, supporting the role of histone PTMs in shaping the chromatin landscape. At the same time, histone PTMs were being linked to cell function, developmental pathways, and cancer pathogenesis. As examples, the *Drosophila* PcG and Trithorax (TrxG) group proteins were originally identified as antagonistic regulators of *HOX* genes during embryonic development [108], and human homologs from both gene groups were quickly linked to disease.

**Table 1.** Chromatin reader domains grouped by histone PTM class

Chromatin reader domains		
Methyl-lysine reader	Example (PTM specificity): Function	References
BAH (bromo-adjacent homology)	ORC1 (H4K20me2): DNA replication licensing	[115]
PWWP (Pro-Trp-Trp-Pro)	DNMT3A (H3K36me2/me3): *de novo* DNA methylation	[116]
Tudor	PHF1 (H3K36me3): DNA damage response	[117]
Tandem Tudor	53BP1 (H4K20me2): DNA damage response	[118]
Chromodomain	HP1 (H3K9me3): heterochromatin structure	[99–101]
WD40	EED (H3K27me3): part of PRC2 complex, gene repression	[119]
PHD (plant homeodomain)	ING2 (H3K4me2/me3): gene silencing	[120]
Ankyrin repeats	G9a/GLP (H3K9me1/me2): gene silencing	[121]
ADD	ATRX (H3K9me3): chromatin remodeling	[122,123]
**Acyl-lysine reader**	**Example (PTM specificity): Function**	**Reference**
Bromodomain	p300/PCAF (H3Kac/H4Kac): gene activation	[93]
Tandem bromodomain	TAF1 (H4Kac, bt, cr): part of TFIID, transcription initiation	[124]
YEATS	AF9 (H3Kac, cr): gene activation	[125]
Tandem PHD	DPF3b (H3K14ac): chromatin remodeling, gene activation	[126,127]
**Phosphorylation reader**	**Example (PTM specificity): Function**	**Reference**
14-3-3	14-3-3ζ (H3S10ph): transcriptional elongation	[128]
BIR	Survivin (H2T3ph): supports mitosis	[129]
Tandem BRCT	MDC1 (γH2AX): crucial to DNA damage response	[130]
**Methyl-arginine reader**	**Example (PTM specificity): Function**	**Reference**
Tudor	TDRD3 (H3R17me2a, H4R3me2a): gene activation	[131]
WD40	WDR5 (H3R2me2s): supports open chromatin structure	[132]

This table is non-exhaustive, but instead demonstrates the breadth of histone PTM interactions and functions, with key examples and references given for each domain subtype.

ac, acetylation; bt, butyrylation; cr, crotonylation; me2a, asymmetric methylation; me2s, symmetric methylation; ph, phosphorylation.

The human *Trithorax* gene *MLL1* (mixed-lineage leukemia 1; also known as *KMT2A*) was first identified in gene translocations associated with aggressive lymphoid and myeloid leukemia [133–135]. *MLL1* fusions are present in 70–80% of infant acute lymphoblastic leukemia, and frequently detected in adult-onset acute myeloid leukemia [136,137]. Unlike *E2A-PBX1* and *TEL-AML1* rearrangements, which could be used to predict therapeutic responses, leukemias with *MLL1* fusions poorly responded to most chemotherapies and had a dire prognosis [138]. Research on the *S. cerevisiae* homolog Set1 revealed its H3K4-specific methyltransferase activity [139–141], but the role of MLL1 in leukemia remained unclear. In 2002, the Hess group confirmed human MLL1 as an H3K4 methyltransferase, and showed the MLL1 SET domain was sufficient for *HOX* gene activation [142]. The same year, several groups reported that leukemias with *MLL1* rearrangements often lead to *HOX* gene overexpression [143,144]. Understanding how *MLL1* rearrangement and fusion impacts gene expression has proven key to developing therapeutics for these challenging leukemias [137].

By 2000 the mutation and dysregulation of human *Polycomb* genes had also been linked to several types of lymphoma [145–147]. Following the discovery of EZH2 as an H3K27 methyltransferase [109–111], it was almost immediately linked to aberrant gene regulation in prostate cancer [148]. Later reports established EZH2 overexpression as a common feature of many cancers, including breast, colon, and bladder [149–151]. With these growing hints to the importance of the chromatin landscape in disease pathology, scientists looked for new strategies to interrogate the PTM binding specificity of chromatin-associated proteins.

## Nucleosomes vs. histone peptides: developing representative chromatin substrates

When it came to defining the interactome of histone PTMs and readers, scientists had two options: *nucleosomes* and *histone peptides*. Although defined protocols for recombinant nucleosomes were available [41,42], such methods were difficult, time-consuming, and generated low yields. Histone peptides, in contrast, were commercially available by the 1990s and easily integrated to existing assays, making them the clear reagent of choice.

Were histone peptides a fully representative substrate? From the outset, researchers had concerns. Chromatin regulatory proteins leverage multiple enzymatic and binding domains to impart function. Peptides drastically limit this complexity, making them unsuitable for studying electrostatic effects of histone acetylation, reader interactions with DNA or the H2A/H2B acidic patch, or the impact of combinatorial PTM *in trans*. By 2003, researchers had identified multiple ‘nucleosome-specific’ KMTs, including EZH2 (targets H3K27; [109–111]), DOT1L (H3K79; [105,152–154]), SETD2 (H3K36; [155]), and SETD8 (H4K20; [156,157]), and discovered several mechanisms requiring histone PTMs in *trans* [103–106]. Given this knowledge, why did the field favor histone peptides?

It was perhaps, in part, a misconception. The early 2000s saw a robust focus on histone PTMs and reader proteins. This PTM-centric era was supported by the prevailing nucleosome model, which depicted N-terminal histone tails protruding from the nucleosome core, suggesting independent function (Figure 2) [55,56]. Thus, one could logically interrogate PTM binding using modified histone peptides. Notably, this model ignored the literature that histone tails associate with DNA and can be modulated by acetylation state (see discussion above). Nevertheless, many scientists used isolated binding and/or enzymatic domains alongside peptide substrates: this reductionism had the ever-present potential to miss or misconstrue data.

Here, we discuss the rise of histone peptide substrates and their essential role in histone PTM discovery and analysis. We also review the challenges and steady improvements in modified nucleosome technologies, which helped demonstrate the significance of higher order context in chromatin biology.

### Deciphering the histone code with modified histone peptides

The automation of Solid-Phase Peptide Synthesis (SPSS), originally developed by Merrifield and colleagues in the 1960s, supported major transformations in protein biochemistry and drug development [33]. By the 1990s SPSS generated fully defined, homogeneous histone peptides at accessible scale and cost, and provided a critical juncture in the characterization of histone tail interactions. Finally, researchers were freed from the limitations of heterogeneous histones purified from cell extracts. Histone peptides provided more reliable data and improved control over experimental design. Importantly, the syntheses also enabled the incorporation of site-specific PTMs, including combinatorials [33].

Histone peptides were instrumental to the creation and validation of PTM antibodies starting in the late 1980s [158–160]. However, they were perhaps most widely used to characterize the binding preference of histone reader domains. Biotinylated peptides were used to fish novel binders from whole cell extracts, or to examine the capability of epitope-tagged reader domains (WT or mutated to explore structural predictions) [161–163]. The application of synthetic peptide array (SPOT) technology, which parallelized direct synthesis on cellulose membranes, expanded the diversity that could be screened in a single array, allowing greater throughput and versatility (Figure 1B) [164–166].

By the mid-2000s, readers had been identified within all categories of histone modifiers, chromatin remodelers, and accompanying complexes [167]. In a landmark study by the Structural Genomics Consortium (SGC), histone peptides were used to probe the binding specificity of 30 different bromodomains and generate [reader : ligand] structures, providing a comprehensive analysis of the family [168]. Histone peptide arrays were also used to interrogate the binding preferences of the growing number of methyl-lysine readers [120,169–172], as well as the newly identified YEATs domains, shown to prefer bulkier crotonyl acyl groups over the more commonly studied acetylation marks [125,173]. The fact that different readers from the same domain class could have distinct PTM binding specificity — and thus disparate functional outcomes — was an important advance in our understanding of a histone code chromatin biology.

### Assembling nucleosomes with modifications

Despite these peptide-derived insights, more effective studies of chromatin interactors require physiological substrates. Only nucleosomes enable dissections of histone charge alterations, tail accessibility, and potential multivalent interactions with DNA, the H2A/H2B acidic patch, and/or other PTMs (see Figure 2). Though it was feasible to make recombinant nucleosomes by the late 1990s [41,42], the addition of defined PTMs was still a struggle. When attempted, the enzymatic modification of nucleosomes was often inefficient and yielded heterogeneous product (e.g. variable levels of me0, me1, me2 and me3 at a given lysine). Thus, the field focused on genetic, biochemical, and synthetic strategies to add PTMs to histones prior to nucleosome reconstitution [33].

In amber codon suppression systems, bacterial strains are genetically engineered to insert an unnatural amino acid (e.g. Kac) when encountering an amber stop codon (UAG) substituted at a target residue. This is achieved by co-expressing the mutated histone with a modified tRNA (carrying a Kac) and orthogonal aminoacyl-tRNA synthetase (to load the tRNA) [174–176]. Amber suppression has been used to study histone lysine acetylation [175], crotonylation [177], and methylation [176], as well as serine phosphorylation [178,179]. However, the approach has various shortcomings that has precluded widespread adoption. Many modified histones yield minimal product, or show variable incorporation of the unnatural amino acid [180]. Perhaps most notably, amber suppression is limited to a single PTM type, restricting the study of combinatorials.

Methyl-lysine analogs, or MLAs, were specifically developed to study histone methyl-lysine PTMs [181]. The method substitutes cysteine for lysine at the desired residue, which can be alkylated *in vitro* to form a methyl-amino-cysteine: structurally and chemically analogous to methyl-lysine. Though several chromatin binding domains and enzymes have been characterized using MLAs [182–190], great care needs to be taken when interpreting results. The Ruthenburg group found that MLAs introduce steric restrictions that can dramatically alter reader binding affinity relative to native PTMs [191]. On comparing MLAs and native PTMs in histone peptide binding assays, data agreed for the ING1-PHD (to H3K4me3), but not for the HP1 CD (to H3K9me3) [182]. Further undermining their usefulness, MLAs cannot be used as enzyme substrates, such as to examine if the MLL1-C KMT can convert H3K4me2 to K4me3 [192].

The technique with perhaps the broadest application to modified nucleosome development is native chemical ligation (NCL), which can be applied to all histone N-terminal tails. In brief, the method uses *trans*-thioesterification to ligate a synthetic histone peptide (containing one or more PTMs) to an appropriate histone fragment to reconstitute full-length histone [193–195]. In 2003, Shogren-Knaak and Peterson developed their first modified histone (H3S10ph) by NCL and successfully incorporated same into nucleosomes to characterize the Gcn5 KAT [195]. In original NCL methods, the modified histone was scarred by mutation at the ligation site [194]. However, they can be made scarless by judicious planning, where an N-terminal alanine on the histone core is mutated to cysteine, used for the ligation step, and desulfurized to native alanine, thus yielding a fully native histone [196–199]. NCL has been optimized for scaled production, making it feasible to generate milligram-levels of PTM-defined histones for nucleosome assembly [200]. Furthermore, approaches have been developed such that any histone residue is now accessible to yield single or combinatorial PTMs across diverse classes (e.g. arginine citrullination or methylation; lysine acylation, methylation, or ubiquitination; serine and threonine phosphorylation).

In the last 5 years, improvements in PTM-defined nucleosome development have coincided with massive advances in protein purification, cryogenic electron microscopy (cryo-EM), nuclear magnetic resonance (NMR), and mass spectrometry. At the same time, the field has made incredible leaps in chromatin mapping technologies, particularly with the development of high-resolution, low-input epigenomic assays (e.g. CUT&RUN and CUT&Tag; see [201–203]). Below we highlight the impact of these innovations in the study of disease-relevant chromatin writers, readers, and erasers. For additional insight, we recommend several excellent recent reviews [107,204–206].

## The nucleosome context defines multivalent chromatin binding mechanisms

By the 2000s, the histone tails and many reader proteins were known to interact with nucleosomal DNA and/or the acidic patch. More recent NMR studies suggested unmodified histone tails ‘collapse’ onto the globular core by interactions with nucleosomal DNA [57,58]. This provides a stark contrast with the classical graphic of extended tails (Figure 2A,B) and suggests that reader proteins/modifying enzymes navigate a complex molecular landscape to engage their target. Emerging evidence shows how various PTMs can alter histone tail contacts with the nucleosome core, refine protein binding specificity, and facilitate multivalent interactions: all insights that are off-limits to peptide-based studies.

### Histone acetylation regulates interactions with nucleosomal DNA

Research on BPTF illustrates how the [DNA : histone tail] interface can regulate complex, combinatorial chromatin interactions. BPTF is a subunit of the NURF chromatin remodeling complex, and linked to multiple human cancers [207]. The original characterization of BPTF by the Allis group uncovered a C-terminal tandem reader domain, comprising a PHD finger, which bound H3K4me3 peptides [171,208], and a bromodomain, which bound acetylated H4 peptides [209]. Of note, other chromatin-associated proteins with multivalent reader engagement were discovered at this time, including the dual PHD finger and CDs of the Rpd3S KDAC complex [210] and the double bromodomain of TAFII250 [124]. However, the discovery of a protein that appeared to recognize histone PTMs *in trans* provided an exciting example of the complexity that might operate.

**Figure 4.**
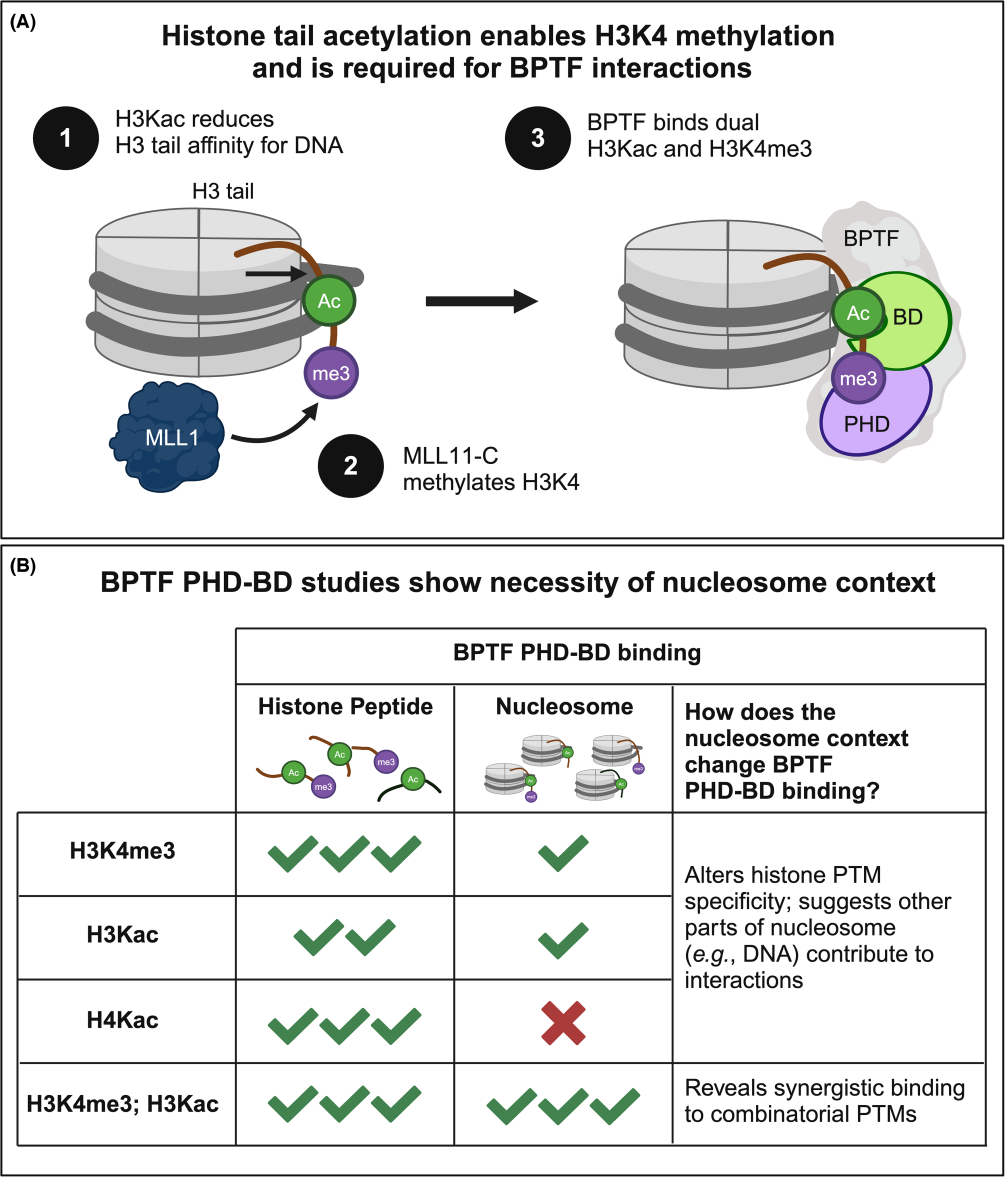
Studies of MLL1-C lysine methyltransferase complex and the BPTF PHD-BD tandem reader illustrate the importance of nucleosome context for chromatin research [59,192]. (**A**) A coherent mechanism links H3 tail acetylation, H3K4 methylation, and BPTF binding. Acetylation reduces H3 tail affinity for nucleosomal DNA, allowing MLL1-C to effectively catalyze H3K4me3 in *cis*. The BPTF PHD-BD tandem reader requires both H3K4me3 and H3K14ac/H3K18ac in *cis* for synergistic binding. (**B**) Experimental results demonstrate key differences in BPTF binding between peptide and nucleosome substrates. Interestingly, though the Kac and Kme cross-talk occurs on the same nucleosomal H3 tail (i.e. *cis* vs. *trans*), this interplay is not detected on peptides. Figure created with BioRender.

To truly appreciate combinatorial chromatin interactions, it is essential to understand how various nucleosome features may be involved. A recent study re-evaluated the PTM preference of BPTF PHD–BD on libraries of fully defined peptides and nucleosomes in parallel [59] — an unbiased approach versus the more common of using positive peptide data to select nucleosomes for further study [211]. Results with peptides replicated the literature, with PHD–BD binding H3K4me3 and poly-acetylated H4 (H4Kac) or H3 (H3Kac), though no indication of co-operative engagement to the H3K4me3Kac combinatorial (Figure 4B). However, on nucleosomes, PHD–BD lost engagement with H4Kac and showed >20-fold increased binding to H3K4me3Kac over either PTM class alone (Figure 4B) [59]. This synergy indicates the PHD–BD tandem was more capable than predicted from either individual reader. The whole was greater than the sum of the parts — an example where the reductionist peptide approach had misled.

Why did the BPTF tandem reader show reduced affinity on nucleosomes vs. histone peptides, even losing the ability to bind H4Kac? NMR suggests this is largely due to the default histone tail associations with DNA, which sets up a competitive landscape for reader engagement [59]. Acetylation weakens histone H3 tail contacts with DNA, which enhances BPTF PHD interactions with *cis* H3K4me3 [57]. In contrast, the H4 tail strongly associates with nucleosomal DNA and makes extensive contacts with the H2A/H2B acidic patch (Figure 2B), even in the presence of H4Kac, which occludes BPTF BD binding. However, BRD4 BD1, a confirmed H4Kac reader, can also bind DNA [212,213], and this competition may help disengage the H4 tail from the nucleosome core. Such reader mechanisms clearly demonstrate the essential role of nucleosomes in shaping chromatin interactions.

### Histone acetylation modulates H3K4me3 reader and writer activity

The study of BPTF revealed key links between H3 acetylation, histone tail accessibility, and H3K4me3 reader interactions. Of note coincident H3 tail acetylation and K4 methylation was one of the first combinatorial histone PTMs described, and its high evolutionary conservation [214,215] suggested important function. Yet decades of research had failed to definitively explain the relationship. Could acetylation-induced changes in nucleosome structure be a prerequisite for H3K4me3?

A 2023 study showed the H3K4 methyltransferase MLL1-C is more effective at generating H3K4me3 in the presence of H3 acetylation, but only in the nucleosome context [192]. The availability of heterotypic nucleosomes, with a single modifiable H3K4 *cis* or *trans* to H3 acetylation, showed the former to be the optimal MLL1-C substrate, supporting a model where charge neutralization promotes histone tail accessibility. Of note, these results define the product of MLL1-C activity as the optimal target for BPTF PHD–BD engagement: H3K4me3Kac in *cis* (Figure 4A). Nucleosome context is a central regulator of this pathway, with peptides completely missing the PTM interplay [192].

## Nucleosome context redirects inhibitor development

Despite widespread interest in drugging histone modifying enzymes and reader proteins for cancer therapy, surprisingly few programs have shown success. Common problems include poor inhibitor selectivity, due to highly conserved protein domains, and low efficacy, due in part to competing high-affinity *in vivo* interactions [216,217]. However substrate choice can also contribute to project derailment, as illustrated in studies of SETD8, the only known H4K20 mono-methyltransferase [156,157], and NSD2, a H3K36 KMT [218]. Though both enzymes were known to be ‘nucleosome-specific,’ histone peptides were still used to develop inhibitors, yielding compounds with poor *in vivo* potency. The application of nucleosomes gives insight to catalytic activity and multivalent binding interactions, and should dramatically alter the path of future inhibitor campaigns.

### Challenges with SETD8 inhibitors

The first identified H4 lysine methyltransferase was SETD8 (KMT5, PR-Set7), which generates H4K20me1 [156,157] and has essential roles in maintaining genome integrity [219–224]. SETD8 is regulated in a cell-cycle specific manner by DNA replication protein PCNA, which targets the methyltransferase for proteasomal degradation [225–228]. The discovery that SETD8 also methylates p53 [229] and is overexpressed in various cancers [230] flagged the enzyme as a promising therapeutic candidate.

The first SETD8 inhibitor was SAM-cofactor (S-adenosyl methionine) competitive nahuoic acid A, a natural product from a *Streptomyces* sp. isolated from marine sediment in 2012 [231]. The substrate-competitive compound UNC0379 was then reported in 2014, that showed µM potency, strong enzyme selectivity [232], and dose-dependent H4K20me1 reduction in neuroblastoma cells [233]. Importantly, the tool compound had been advanced using the isolated SET domain of SETD8 and H4 peptide substrates. This was a standard approach at the time, due to the relatively low cost of tagged protein domains and histone peptides, as well as their compatibility with high-throughput screening strategies. Although nucleosomes were available, they were comparably expensive and necessitated extensive workflow optimization. However, further research into SETD8 provided a salutary lesson that the histone peptide shortcut could yield sub-optimal drug candidates.

In 2016, Song Tan's group published the structure of full-length SETD8 bound to a nucleosome, which revealed multivalent contacts with nucleosomal DNA and the H2A/H2B acidic patch [234]. These interactions position the enzyme for catalytic activity and occlude PCNA binding [225–228]. Later that same year, Eli Lilly showed SETD8 has a ∼10 000-fold higher affinity for nucleosomes compared with peptides; that nucleosomes improve SAM-cofactor binding to the enzyme; and further, that DNA binding by the SETD8 N-terminus may change the architecture of the substrate binding pocket [235]. Such an understanding of enzyme function should be an integral element of inhibitor development campaigns. As above, UNC0379 resulted from a pipeline that selected for inhibition of the SETD8 catalytic domain on histone peptides. However, it is a poor inhibitor of the full-length enzyme and nucleosome context, and may thus have an inherently limited *in vivo* efficacy. In contrast, starting with the physiological enzyme and substrate can yield novel chemical matter with greater long-term potential [235].

### The nucleosome context transforms NSD2 inhibitor programs

*NSD2* (*MMSET*, *WHSC1*) was first identified by t(4;14) gene translocations in multiple myeloma [236,237], and later defined as a nucleosome-specific KMT for H3K36me2 [218]. Activating mutations and/or overexpression directly contributes to gene activation, cell proliferation, and oncogenesis, making NSD2 a desirable therapeutic target. Drug development studies typically target catalytic domains, which have had high rates of success for many enzyme classes, particularly kinases [238]. However, in the case of NSD2, targeting its catalytic SET domain was challenged by the requirement for nucleosome substrates, which were still difficult to produce and less compatible with high-throughput screening approaches. Instead, researchers targeted the NSD2-PWWP1 reader domain, which binds H3K36me2 and stabilizes NSD2 on chromatin, supporting transcriptional activation [239–241]. Screening with the isolated PWWP1 domain and H3K36me2 peptides led to the identification of multiple candidates, though most lacked activity *in vivo*.

Could nucleosomes provide additional insight? PWWP domains interact with both methyl-lysine and nucleosomal DNA [242,243], so higher order context is almost certainly required to develop an effective inhibitor. Moreover, NSD2 contains multiple reader domains, so full-length protein may also be needed to accurately model multivalent binding. A recent study from the SGC employed H3K36me2 nucleosomes to develop the NSD2-PWWP1 inhibitor UNC6934, which showed high selectivity against a collection of methyl-lysine binding domains and striking potency (IC_50_ < 100 nM) relative to other NSD2 inhibitors [244]. Of note, while UNC6934 can successfully block the binding of full-length NSD2 to nucleosomes *in vitro*, it induces nucleolar accumulation of NSD2 in cells, indicating complex multivalent engagement pathways [244]. Fortunately, the UNC6934 campaign had paralleled progress with a new tool of modern drug developers: ‘targeted protein degraders.’ These link target-specific compounds to domains that recruit the protein degradation machinery, promoting target destruction [245]. With this approach, UNC6934 has proven rather useful, leading to two successful NSD2-targeted degraders, including UNC8153 [246,247]. The addition of UNC8153 to multiple myeloma lines resulted in dose- and time-dependent decreases in NSD2 and H3K36me2, as well as changes in cancer-related phenotypes [246]. Such approaches might be able to overcome the high barrier of effectively inhibiting multivalent engagement and enable a new era of epigenetic drugs.

## Histone PTM antibodies: assay-specific validation matters

Antibodies to histone PTMs are essential for chromatin research. In many of the above studies antibodies were used to relate site-specific enzyme activity with reader engagement. They also supported the genomic mapping of PTM enrichment, as the field moved from single location studies to microarrays to next-generation sequencing technologies [248,249].

The development of anti-histone PTM antibodies has always been a challenge [250–253], as these reagents must distinguish highly related states: e.g. H3K4me1, me2, or me3 from each other and methylations at other residues. Furthermore, and in an ideal world that is rarely effectively explored, the antibody should bind its PTM independent of any *in vivo* combinatorial context. Finally, for genomic applications (e.g. Chromatin ImmunoPrecipitation; ChIP) the antibody must effectively engage its target in the context of higher order structures, including the nucleosome and any associated proteins.

Antibody quality (i.e. target specificity, affinity, and reliability) has proven a major concern across all biomedical research [254], and those to histone PTMs are no exception [250–253]. Conventional wisdom suggested production processes may be to blame. For decades, the industry standard was polyclonal sera, which describes a mixture of antibodies generated from an immune response. Though fast and inexpensive, polyclonals often show high lot-to-lot variability, leading the field to shift to monoclonal antibodies derived from a single cloned parent cell. Once in hand, these can be produced at will, and should show minimal lot variability. Yet, despite some improvements, the switch to monoclonals did not solve the problem. Could antibody validation techniques also be a culprit?

Antibodies to histone PTMs are raised against peptide immunogens, followed by extensive peptide-based screening and confirmation — they are rarely tested in higher order context. To determine if this strategy accurately predicts ChIP performance, >50 antibodies to H3K4methyl states were examined by histone peptide microarray (the field gold standard) and ICe ChIP (Internal Standard Calibrated ChIP) [255]. The latter improved on the usual approach by incorporating DNA-barcoded, PTM-defined nucleosomes as spike-ins for *in situ* testing of antibody specificity and efficiency in ChIP [256]. The resulting data showed no predictive relationship between the two approaches. Further many of the most highly cited antibodies to H3K4me3 profoundly cross-reacted with H3K4me2 [255], a more abundant *in vivo* target [257]. Likely, initial ChIP datasets had been created with such cross-reactive reagents, and all succeeding had to deliver the same peak structures and material yields or fail to be adopted, and peptide array testing could not distinguish the good from the bad. Scientists at *EpiCypher* have expanded this study, developing nucleosome spike-in controls (SNAP-ChIP®) to screen >400 commercial antibodies to lysine methyl and acyl PTMs, and determined that >70% of those used for genomic mapping fail one or both specificity and efficiency metrics (<20% cross-reactivity and >5% target recovery: manuscript in preparation and chromatinantibodies.com). Combined, this body of work demonstrates that peptide-based PTM antibody validation has provided false confidence, and *in situ* nucleosome standards are required to identify reagents capable of genomic mapping.

## Nucleosome context enables detailed structural analyses

Similar to other enzyme classes (e.g. kinases), the activity of chromatin-modifying enzymes can be regulated by conformational changes that enhance or impede substrate binding. A powerful method to define these [enzyme : substrate] interactions is single particle *cryo-EM*, which can generate three-dimensional models of macromolecular structures without the need for crystallization [258]. Cryo-EM studies require the researcher to consider multiple factors, including histone PTM flexibility, complex stability, transient interactions, or distinct stages of catalysis (often explored/stabilized by catalytic site mutations, cofactor analogues, or enzymatic byproducts) [259–263]. Perhaps more than any other approach, cryo-EM has demonstrated the mechanistic insight that only nucleosome context can provide.

### Allosteric changes require nucleosomes to study

The highly conserved H3K79 methyltransferase DOT1L was the first KMT identified that targets the histone globular core, and the only one without a SET catalytic domain [152,153]. H3K79 methylation is enriched across actively expressed genes and thought to support transcriptional elongation, though the precise mechanism is poorly understood. DOT1L also regulates telomere silencing, DNA repair, and cell division, while mouse models indicate critical roles in hematopoiesis and embryonic development [264–268]. In *MLL*-rearranged leukemias, DOT1L interacts with the fusions to activate transcription and drive cancer development [269,270]. As a result, DOT1L is a popular therapeutic target, with several inhibitors in Phase I clinical trials [271–273].

**Figure 5.**
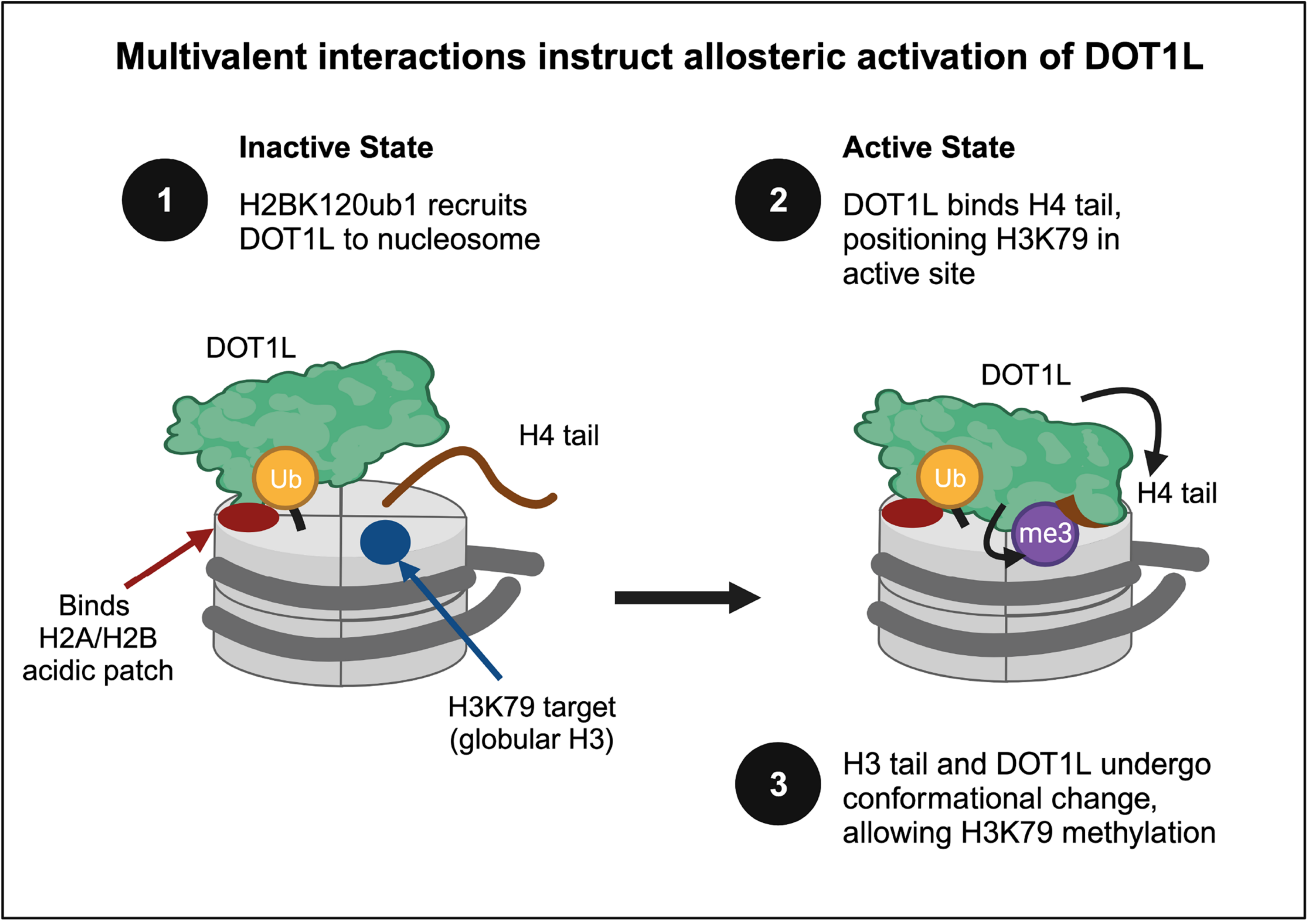
Allosteric activation of DOT1L methyltransferase requires multivalent engagement with a H2BK120ub1 nucleosome substrate [275–278]. DOT1L recruitment to a H2BK120ub1 nucleosome is stabilized through interactions with the H2A/H2B acidic patch. To convert from ‘resting’ to ‘active’ state, DOT1L binds the H4 tail (brown), which drives conformational changes that place H3K79 within the enzyme active site. Adapted from [277]. Figure created with BioRender.

Shortly after its discovery, researchers found that H2BK120ub1 stimulated human DOT1L activity *in vivo*, most likely by an allosteric mechanism [105,106]. The next decades saw huge improvements in cryo-EM resolution [274] and the availability of PTM-defined nucleosome substrates, and in 2019 multiple groups converged on the DOT1L binding mechanism [275–279]. As depicted in Figure 5, DOT1L pivots between ‘poised’ and ‘active’ states, and in each interacts with H2BK120ub1, nucleosomal DNA, and the H2A/H2B acidic patch. The active state positions DOT1L near H3K79 and enables interactions with the H4 tail, leading to conformational changes that place H3K79 within the enzyme active site [277]. DOT1L thus represents a mechanism that involves three core histones, DNA, and conformational changes within the nucleosome globular core. Additional cryo-EM studies have identified a role in this enzymatic pathway for H4K16ac, a PTM that also regulates inter-nucleosomal interactions and chromatin architecture [280].

### Understanding polycomb repressive deubiquitinase deubiquitinase mutations in cancer

As with other monoubiquitination PTMs, the regulation of H2AK119ub1 is central to controlling gene expression pathways. The PTM is removed by the Polycomb repressive deubiquitinase (PR-DUB) complex, which prevents aberrant gene silencing by PRC2 and supports gene activation [281–285]. PR-DUB is a heterodimer of a conserved ubiquitin C-terminal hydrolase (BAP1) and a deubiquitinase adaptor protein (ASXL1, ASXL2, or ASXL3) that recruits and stabilizes the enzyme on chromatin [286]. *BAP1/ASXL1* mutations are common in multiple cancers and are associated with poor outcomes. Indeed, germline mutations in *BAP1* are often characterized as ‘BAP tumor predisposition syndrome,’ because affected patients typically develop early onset, metastatic cancer. Mutations in *ASXL1* may cause gain of function in the PR-DUB complex, supporting altered immune cell differentiation and the development of myeloid leukemia [287–289].

Understanding how PR-DUB regulates H2AK119ub1 and PRC2 signaling is essential to the development of therapeutics targeting this complex. Explaining the mechanism driving PR-DUB substrate specificity, and the impact of cancer associated mutations, is particularly critical. The Armache group applied cryo-EM to generate a high-resolution structure of PR-DUB (BAP1/ASXL1) bound to a H2AK119ub1 nucleosome [290]. Their structure showed that each PR-DUB subunit makes multiple points of contact with the substrate, including H2AK119ub1, DNA, and the H2A/H2B acidic patch. The combined interactions position BAP1 near the ubiquitinated residue to support enzyme activity [290] and help explain the specificity of PR-DUB for H2AK119ub1, as other ubiquitinated residues would be inaccessible in the engaged position. Of note, mutation of the nucleosome interaction sites compromises DUB activity towards a H2AK119ub1 nucleosome, but not to a similarly modified histone peptide. Finally, a range of cancer associated mutations [287–289] within the BAP1/ASXL1 nucleosome-interacting regions also reduce DUB activity on H2AK119ub1 nucleosomes, providing a potential mechanistic explanation for disease development in a subset of patients [290]. So, a single [DUB : nucleosome] cryo-EM complex provides detailed insight to enzyme activity, substrate preference, and human disease, but a peptide is blind to all these aspects: an encapsulation of the importance of physiological substrates in the characterization of chromatin-modifying complexes.

## Context matters: future technologies in histone PTM research

Despite these remarkable advances in our understanding of histone PTM biology, so much remains to be done [291]. Specifically, how do chromatin regulators work together inside a cell to co-ordinate downstream events? How are these processes regulated across different cell types, and dysregulated in disease? And what is the best strategy for leveraging histone PTMs into biomarkers and improved therapeutics? Our current knowledge is limited to some highly detailed *in vitro* structural models and (often) indirect *in vivo* experiments. We now need more controlled studies with appropriately qualified reagents to allow the examination of nucleosomes in the cellular context.

How might this be accomplished? Recent reviews propose innovative strategies, with many focused on single cell analysis [291–295]. Cytometry Time of Flight combined with mass spectrometry is enabling the simultaneous measurement of multiple histone PTMs from single cells [296,297]. Spatial epigenomics provides genome-wide profiles while preserving temporal resolution, revealing complex chromatin states across tissues [298,299]. Ultra-sensitive microscopy techniques are allowing researchers to study chromatin looping and structural changes in real time [293,294]. Finally, Nuc-MS can provide a deeper understanding of how histone variants and PTMs are co-ordinated within nucleosomes (i.e. a nucleosome code) [300,301]. Combined, these advances will continue to expand our knowledge of the function of histone PTMs, connecting the ‘histone code’ to its many regulators, responders, and beyond in regulating cellular function.

## Abbreviations

BD: bromodomain
CD: chromodomain
ChIP: chromatin immunoprecipitation
cryo-EM: cryogenic electron microscopy
KAT: lysine acetyltransferase
KDAC: lysine deacetylase
KDM: lysine demethylase
KMT: lysine methyltransferase
NCL: native chemical ligation
NMR: nuclear magnetic resonance
PR-DUB: Polycomb repressive deubiquitinase
PRC2: Polycomb Repressive Complex
PTMs: post-translational modifications
SGC: Structural Genomics Consortium
